# Integrated Rapid Mapping of Neglected Tropical Diseases in Three States of South Sudan: Survey Findings and Treatment Needs

**DOI:** 10.1371/journal.pone.0052789

**Published:** 2012-12-20

**Authors:** Timothy P. Finn, Barclay T. Stewart, Heidi L. Reid, Nora Petty, Anthony Sabasio, David Oguttu, Mounir Lado, Simon J. Brooker, Jan H. Kolaczinski

**Affiliations:** 1 Malaria Consortium, South Sudan Country Office, Juba, Republic of South Sudan; 2 Department of Disease Control, London School of Hygiene and Tropical Medicine, London, United Kingdom; 3 Medical University of South Carolina, Charleston, South Carolina, United States of America; 4 School of Population Health, University of Queensland, Herston, Queensland, Australia; 5 Vector Control Division, Ministry of Health, Kampala, Uganda; 6 Ministry of Health, Juba, Republic of South Sudan; 7 Kenya Medical Research Institute – Wellcome Trust Research Programme, Nairobi, Kenya; 8 Malaria Consortium, Africa Regional Office, Kampala, Uganda; Universidade Federal de Minas Gerais, Brazil

## Abstract

**Background:**

Integrated rapid mapping to target interventions for schistosomiasis, soil-transmitted helminthiasis (STH) and lymphatic filariasis (LF) is ongoing in South Sudan. From May to September 2010, three states – Unity, Eastern Equatoria and Central Equatoria – were surveyed with the aim of identifying which administrative areas are eligible for mass drug administration (MDA) of preventive chemotherapy (PCT).

**Methods and Principal Findings:**

Payams (third administrative tier) were surveyed for *Schistosoma mansoni, S. haematobium* and STH infections while counties (second administrative tier) were surveyed for LF. Overall, 12,742 children from 193 sites were tested for schistosome and STH infection and, at a subset of 50 sites, 3,980 adults were tested for LF. Either *S. mansoni* or *S. haematobium* or both species were endemic throughout Unity State and occurred in foci in Central and Eastern Equatoria. STH infection was endemic throughout Central Equatoria and the western counties of Eastern Equatoria, while LF was endemic over most of Central- and Eastern Equatoria, but only in selected foci in Unity. All areas identified as STH endemic were co-endemic for schistosomiasis and/or LF.

**Conclusions:**

The distribution and prevalence of major NTDs, particularly schistosomiasis, varies considerably throughout South Sudan. Rapid mapping is therefore important in identifying (co)-endemic areas. The present survey established that across the three surveyed states between 1.2 and 1.4 million individuals are estimated to be eligible for regular MDA with PCT to treat STH and schistosomiasis, respectively, while approximately 1.3 million individuals residing in Central- and Eastern Equatoria are estimated to require MDA for LF.

## Introduction

South Sudan established a national programme for the integrated control of neglected tropical diseases (NTDs) in 2008, with support from the United States Agency for International Development (USAID). The programme targets five key diseases: lymphatic filariasis (LF), onchocerciasis, trachoma, schistosomiasis due to Schistosoma mansoni and S. haematobium, and soil-transmitted helminths (STH: hookworms, Ascaris lumbricoides and Trichuris trichiura). Although a large number of NTDs are thought to be endemic in South Sudan [1], the above five were prioritised because safe and effective preventive chemotherapy (PCT) is available free of charge or at low cost due to drug donation programmes. In areas where more than one of these diseases are endemic, some of the PCT drugs can be safely administered in combination [2].

At programme inception, detailed information on the prevalence and distribution of LF, STH and schistosomiasis was absent or incomplete. An integrated rapid mapping protocol was therefore developed to generate the required data to target mass drug administration (MDA) of PCT to at-risk populations [3]. Onchocerciasis and trachoma were not included in the protocol because the distribution of onchocerciasis had already been mapped by the African Programme for Onchocerciasis Control (APOC) [4], while for trachoma it was felt that the required diagnostic skills and the recommended sampling frame [5] were not compatible with survey methods for helminth infections.

**Figure 1:**
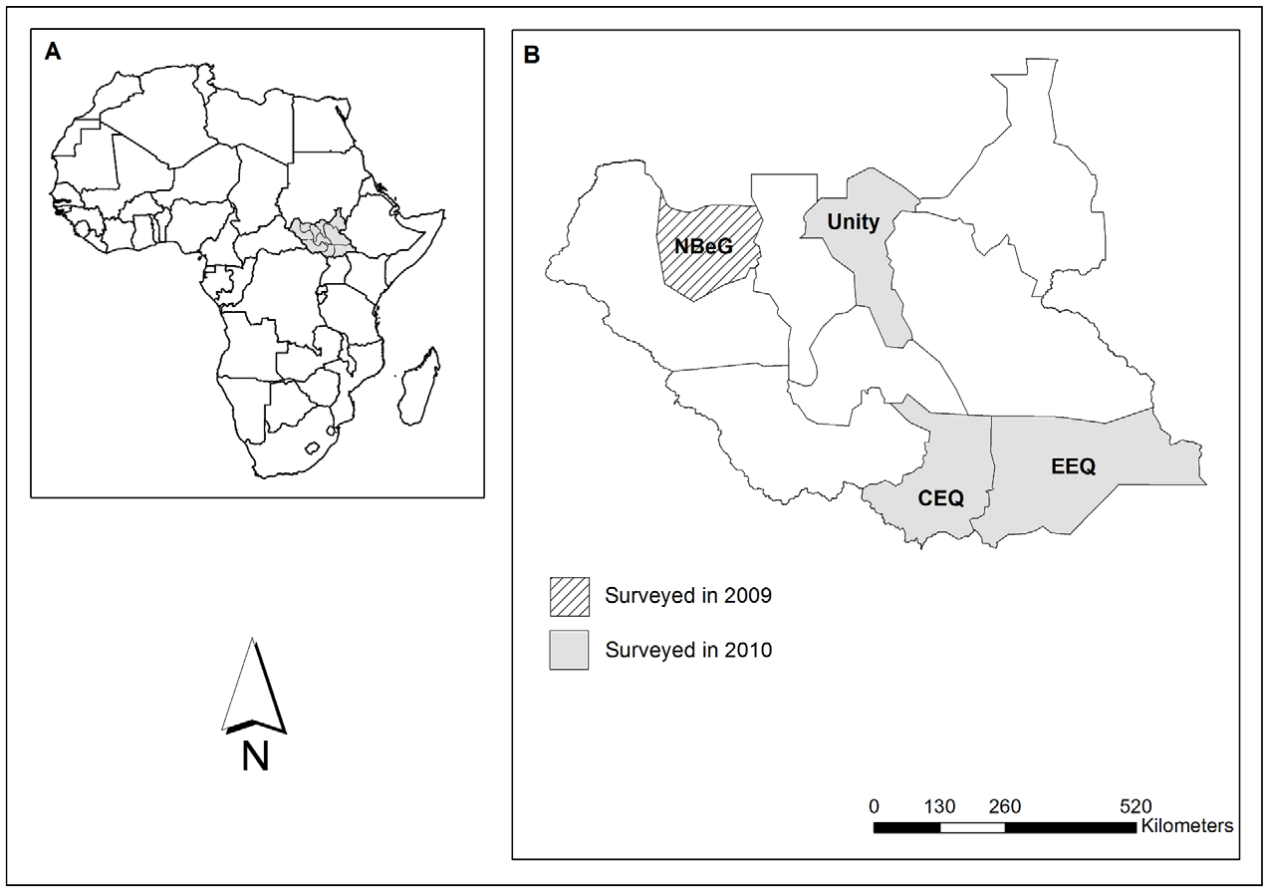
Map of survey areas. A) Africa showing location of South Sudan. B) South Sudan showing location of the three states surveyed in 2010 and referred to in the present manuscript (grey shaded) and Northern Bahr-el-Ghazal (NBeG) surveyed in 2009 (hatched).

One of South Sudan's ten states, Northern Bahr-el-Ghazal, was successfully mapped in 2009 using the integrated survey approach [3]. In 2010, three more states were selected for rapid NTD mapping – Unity, Central Equatoria and Eastern Equatoria – based on their suspected high burden of one or more of the targeted diseases (Figure 1). Here we present the results of this survey and discuss their implications for estimating MDA needs.

## Methods

### Ethical Considerations

The Directorate of Research, Planning and Health System Development of the Ministry of Health (MoH) of the Government of South Sudan (GoSS), as well as the London School of Hygiene and Tropical Medicine (LSHTM), U.K., provided ethical review and approval of the survey protocol. Each respective State MoH, County Health Department and Payam Administration was provided with the survey protocol and a description of the proposed activities, and approval for the survey was granted prior to implementation. The purpose and details of the survey were explained to each head of household or guardian in his/her respective language before s/he was requested to provide written/thumbprint consent for the entire household to participate in the survey. If consent was granted, survey particulars were explained to each household inhabitant who met inclusion criteria. Participants were then asked to provide verbal consent before taking part in the survey. Only those who provided consent were registered and were requested to provide samples. To record verbal consent, the name of each participant providing consent was documented. Individuals who tested positive for schistosome or STH infection were provided with a treatment dose of praziquantel or albendazole, respectively, following WHO recommendations [2]. Participants who tested positive for Wuchereria bancrofti antigen were not treated on site. As advised by the MoH-GoSS, they were informed of their infection and its potential consequences and were referred to the nearest health facility for treatment with ivermectin and albendazole.

### Study Sites

The surveys were conducted from May to September 2010 in Unity, Central- and Eastern Equatoria states. South Sudan has a four-tier administrative structure comprised of states (1^st^), counties (2^nd^), payams (3^rd^) and bomas (4^th^). At the time, the three states consisted of a total of 23 counties and 171 payams, and were inhabited by approximately 2.5 million individuals, accounting for around 32% of South Sudan's population. Twenty-two of the 23 counties and 120 of the 171 payams were surveyed (Table 1). Unity State is home to the Nuer and Dinka ethnic groups, nomadic agro-pastoralists who herd cattle in riverine and low-lying areas during the dry season and grow staple grains during the rainy season. Central- and Eastern Equatoria states are inhabited by more than 19 ethnic groups that conduct agricultural activities in the West and South, or pastoralist activities in the East and North.

**Table 1:** Population by state and number of payams surveyed by state.

State	Population (2008)	Total Number of Counties	Number of Counties Surveyed	Total Number of Payams	Number of Payams Surveyed	Total Number of Villages Sampled
Central Equatoria	1,037,012	6	6	45	37	62
Eastern Equatoria	906,126	8	8	53	31	58
Unity	600,572	9	8	73	52	73
Total	2,543,710	23	22	171	120	193

### Survey Methods

Data were collected following the integrated NTD survey protocol, developed by the MoH-GoSS and Malaria Consortium with financial support from USAID through RTI International and with technical support from the Centers for Disease Control and Prevention (CDC). The protocol has been described in detail elsewhere [3] and was slightly modified to reduce the number of sites to be surveyed for LF, following a detailed costing study [6].

The administrative areas surveyed for *S. mansoni, S. haematobium* and STH were the payams, while counties were surveyed for LF. A convenience sampling methodology was employed to maximise the probability of identifying endemic areas. For schistosomiasis, survey villages were selected based on a combination of proximity to water bodies and anecdotal reports of villages with infected individuals. For LF, survey sites were selected based on anecdotal reports of individuals with lymphoedema and/or hydrocele from county administrative and medical staff. STH infection was assumed to be geographically more homogeneously distributed than schistosomiasis and LF [7]–[9], and selection of sites on the basis of schistosomiasis and LF ecology was therefore considered sufficient to capture the inherent spatial heterogeneity of STH infection. In each accessible payam, a minimum of one and a maximum of three sites were surveyed for *S. mansoni, S. haematobium* and STH, depending on the payam population size. For LF, at least one and up to three sites per county were sampled, if no ICT positive cases were found in the first site and if villages were too small to reach a total sample size of 250 individuals ≥16 years in the first two survey sites [3]. Where more than one survey site was selected per payam, the teams ensured that these were geographically distinct and well separated.

A sample of up to 75 children aged 5 to 15 years was registered in each village for schistosomiasis and STH testing; each child was asked to provide stool and urine samples. For LF, up to 110 individuals aged 16 years and above were requested to provide a blood sample in each study village to be tested for circulating *W. bancrofti* antigen with a immunochromatographic card tests (ICT) (BinaxNOW Filariasis, Inverness Medical, Portland, ME, USA). In sites sampled for LF, data on the presence of Loa loa were collected from each adult registered for ICT testing using the RAPLOA rapid assessment procedure [10]. Individuals were excluded from the survey if they had not lived in the area for at least six months.

All samples were processed during the survey day. Faecal samples were examined in duplicate for S. mansoni and STH ova using Kato-Katz technique within an hour of slide preparation. Urine samples were tested for haematuria using Hemastix reagent strips (Siemens Healthcare Diagnostics, Tarrytown, NY, USA), with positive samples being subsequently examined for S. haematobium using urine filtration and microscopy. Coordinates of each study site were collected using handheld GPS devices (eTrex, Garmin International, Kansas, USA).

A follow-up investigation was conducted in November 2010 in an attempt to locate the two individuals from Unity State that tested positive with the ICT during the June 2010 survey and to establish whether these two individuals harboured active infections with *W. bancrofti*. It was originally planned to re-test both individuals with ICT, to collect spots on calibrated filter paper provided by the Centers for Disease Control and Prevention (CDC) for subsequent laboratory analysis using the Og4C3 enzyme-linked immunosorbent assay (ELISA) for LF antigen [11] in Atlanta, and to compile a detailed history on travel and residency for each case. Furthermore, it was planned to test family and other villagers living in close proximity to the two individuals using ICTs and, if found positive, to collect blood spot samples.

### Data Analysis

Data were double entered into Microsoft Excel 2008 (Redmond, Washington, USA). Range and consistency checks were conducted for all non-string variables. Descriptive statistics and prevalence estimates were calculated using STATA 10 (College Station, Texas, USA). Maps showing the location of the survey sites were created using ArcGIS 9.2 (ESRI, California, USA).

### Data Interpretation

WHO recommends a series of intervention thresholds for the diseases mapped in this study. For STH, communities are classified as endemic if prevalence is ≥20%, or hyperendemic if prevalence is ≥50%. In accordance with WHO guidelines, communities endemic or hyperendemic for STH should be treated with a benzimidazole drug such as albendazole on an annual or biannual schedule, respectively. For schistosomiasis, communities are categorised as endemic or hyperendemic if prevalence is ≥10% or ≥50%, respectively [2]. Endemic or hyperendemic communities should be treated biennially or annually, respectively, using praziquantel.

The threshold for categorization of an area as eligible for MDA of PCT to eliminate LF is 1% infection prevalence [2]. For L. loa, a prevalence of 40% of eye worm history is considered the limit above which communities are considered at high risk for severe adverse events from ivermectin treatment [10]. In areas of high loiasis endemicity, LF treatment should not be undertaken unless the area is co-endemic for onchocerciasis and two or more large-scale rounds of ivermectin distribution for onchocerciasis control have been conducted [12].

National census estimates from 2008 were used to calculate the population eligible for treatment within surveyed administrative areas and the number of treatments required. Given the different treatment schedules for STH (i.e. biannual and annual) and schistosomiasis (annual and biennial) a treatment cycle is one year or two years, respectively. In LF endemic areas, treatment with ivermectin should be conducted annually.

## Results

Overall, 13,588 children from 193 sites were registered to be tested for schistosome and STH infection and 3,986 adults from 50 sites were registered for LF testing. From the children, 12,808 urine samples and 12,303 stool samples were examined, while 3,980 blood samples from adults were tested for W. bancrofti antigen. The median age of children sampled was 8 years (inter-quartile range (IQR): 6–11 years) and 52% were male. Of the adults that provided a blood sample, the median age was 32 years (IQR: 25–45 years) and 33.6% were male (Table 2). A total of 51 payams were not sampled, ten of which in one county that was inaccessible to survey teams (Table 1).

**Table 2:** Characteristics of sampled individuals and summary prevalence estimates from Unity, Central Equatoria and Eastern Equatoria States, South Sudan.

	Schistosome^a^ and STH^b^;			Lymphatic filariasis^c^		
	Unity	Central Equatoria	Eastern Equatoria	Unity	Central Equatoria	Eastern Equatoria
Number sampled; n				1,933	1,069	978
Faecal sample	4,387	3,913	4,003			
Urine sample	4,858	4,104	3,846			
Faecal or urine	5,011	4,208	3,986			
Age (yrs); med. (IQR)	7 (6–10)	10 (7–12)	8 (6–11)	34 (27–48)	30 (24–40)	29 (22–39)
Gender; n (%)						
Male	2,669 (53.3)	2,129 (50.6)	1992 (50.0)	555 (28.7)	460 (43.0)	322 (33.2)
Female	2,342 (46.7)	2,079 (49.4)	1994 (50.0)	1,382 (71.4)	609 (57.0)	645 (66.8)
Infected; %						
Schistosomiasisd,e	26.6 (0–78.4)	22.0 (4–90.9)	14.2 (0–60.6)	-	-	-
*S. haematobium*	16.1 (0–78.4)	6.1 (0–90.9)	0.23 (0–4.2)			
*S. mansoni*	17.4 (0–61.0)	17.8 (1.1–88.7)	16.2 (0–71.1)			
STH	0.5 (0–8.3)	39.7 (34.0–45.5)	23.6 (1.5-67.6)	-	-	-
Hookworm sp.	0.3 (0–4.5)	38.9 (7–81.9)	22.2 (0–67.1)			
*A. lumbricoides*	0 (0–0.8)	0	1.1 (0–12.1)			
*T. trichiura*	0.3 (0–8.3)	1.3 (0–40.6)	2.3 (0–13.0)			
LF f	-	-	-	0.1 (0–0.5)	2.2 (0.5–8.1)	3.5 (0.7–9.7)

a Defined as microscopy positive for eggs of *Schistosoma mansoni* or *S. haematobium* in urine and/or stool sample.

b Defined a microscopy positive for eggs of *Ascaris lumbricoides*, *Trichuris trichiura*, and/or hookworms.

c Defined as immunochromatographic test (ICT) positive.

d Tested for either or both *S. haematobium* or *S.mansoni* (i.e. returned either or both faecal or urine samples).

e Prevalence is for state, range is by payam.

f Prevalence is for state, range is by county.

The prevalence of schistosome infection (*S. mansoni* and/or *S. haematobium*) was 26.6%, 22.0% and 14.2% in Unity, Central Equatoria and Eastern Equatoria, respectively. The overall prevalence of S. haematobium in the three states was consistently lower than that of S. mansoni (Table 3). There was marked variation in prevalence by both study site (ranging from 0% to 90.9%) and payam (0%–78.4%, 4.0–90.9% and 0.0%–60.6% in Unity, Central Equatoria and Eastern Equatoria, respectively). Using WHO recommended MDA thresholds, 39/52 (75%), 29/37 (78%), and 13/31 (42%) of surveyed payams in Unity, Central- and Eastern Equatoria qualify for annual or biennial MDA of PCT to treat schistosomiasis (Table 4). Over a two-year treatment cycle approximately 1.4 million praziquantel treatments would be required.

**Table 3:** Number of payams, individuals and doses for MDA of PCT for schistosomiasis over a period of two years in Unity, Central Equatoria and Eastern Equatoria States.

State	Biennial 10–50%		Annual >50%		Tx's PZQ to cover target population^a^
	Payams	At risk	Payams	At risk	
Unity	28	238,263	11	118,825	380,730
Central Equatoria	27	678,152	2	151,078	784,246
Eastern Equatoria	11	218,925	2	49,563	254,441
Totals	66	1,135,340	15	319,466	1,419,418

a Tx's = Treatments; PZQ = Praziquantel; the target population is approximately 80% of the population at risk; tablets per treatment of praziquantel are calculated by height e.g. 1 tablet given to a child between 94 and 110 cm, 5 tablets for an adult >178 cm adults etc. with the average dose given across the target population 3 tablets.

**Table 4:** Number of payams, individuals and doses required for MDA of PCT for soil-transmitted helminthiasis over a period of one year in Unity, Central Equatoria and Eastern Equatoria.

State	Annual 20–50%		Biannual >50%		Tx's ALB to cover target population^a^
	Payams	At risk	Payams	At risk	
Unity	0	0	0	0	0
Central Equatoria	17	485,249	15	274,475	930,779
Eastern Equatoria	10	164,800	4	72,546	278,903
Totals	17	650,049	19	347,021	1,209,682

a Tx's = Treatments; ALB = Albendazole; the target population is approximately 90% of the total population; infants between one and two years are given half a tablet per treatment while the remaining target population above two years receive one tablet per treatment.

**Figure 2:**
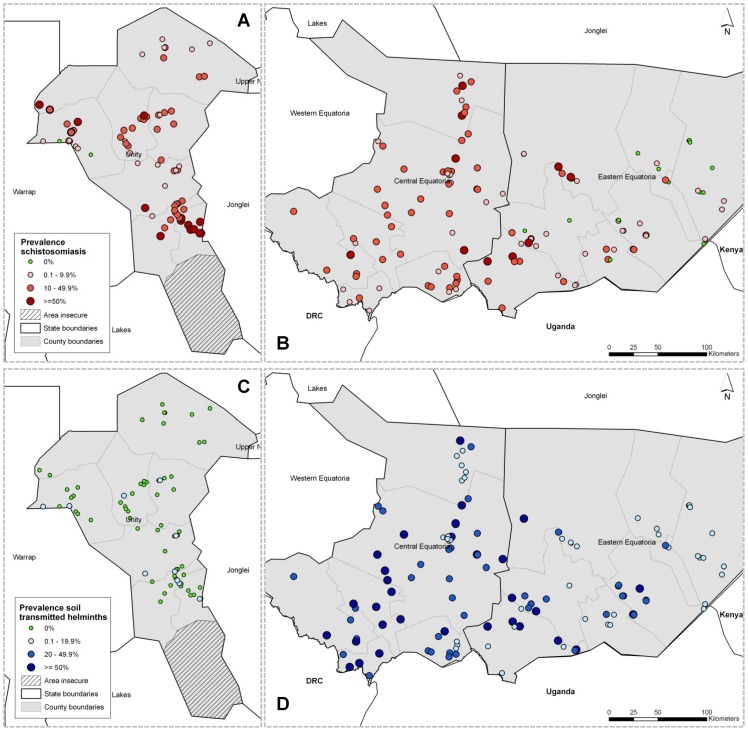
Location of survey areas and sites, and prevalence of STH and SCH infection at each site. A) Map of Unity showing prevalence of SCH infection (*S. mansoni* and/or *S. haematobium*) at each survey site, B) Map of Central Equatoria and Eastern Equatoria states showing schistosomiasis prevalence at each survey site, C) Map of Unity State showing prevalence of STH infection at each survey site, D) Map of Central Equatoria and Eastern Equatoria states showing prevalence of STH infection at each survey site.

Overall, 20.5% of sampled children were infected with at least one STH species. Hookworm was most common, accounting for 92.3% of STH infections across the three states. The overall prevalence of *A. lumbricoides* and *T. trichiura* was 0.37% and 1.27%, respectively. Prevalence of STH infection varied considerably in space (Figure 2): STH infection was much more common in the South of the country, with 32 of 37 (87%) surveyed payams in Central Equatoria and many of the payams in the western part of Eastern Equatoria exceeding WHO recommended MDA thresholds. Unity State, as well as the eastern part of Eastern Equatoria, were not endemic. Based on WHO guidelines, an estimated 1.2 million PCT treatments for STH will be required in the two endemic states over a one-year treatment cycle (Table 4).

Only two individuals in Unity State were identified as positive for circulating *W. bancrofti* antigen using the ICT test; these were resident in separate villages (Pangook and Thorbokui) in different counties (Abiemnhom and Mayendit, respectively) approximately 170 km apart. During a follow up investigation conducted in November 2010, only one of the two positive individuals could be located. The individual, as well as one family member and 10 neighbours (>15 years old), were tested with ICT tests but were all found to be negative for circulating *W. bancrofti* antigen. The individual reported that he had lived in Pangook since birth, and had only made brief visits to neighbouring Mayom and Rubkona counties. Subsequent ELISA analysis of the blood sample by CDC did, however, show that he was clearly positive for *W. bancrofti* antigen. Nevertheless, Mayendit was not considered to be LF endemic, as the prevalence of ICT positive individuals was below 1%. Family members of the second ICT positive individual, who could not be located, were tested and found to be ICT negative. With respect to Abiemnhom, the county was not considered to be LF endemic, as the prevalence of ICT positive individuals (including the unconfirmed individual) was below 1%. No ICT positive individuals were identified in all other counties and Unity State, as a whole, should not be considered LF endemic.

**Figure 3:**
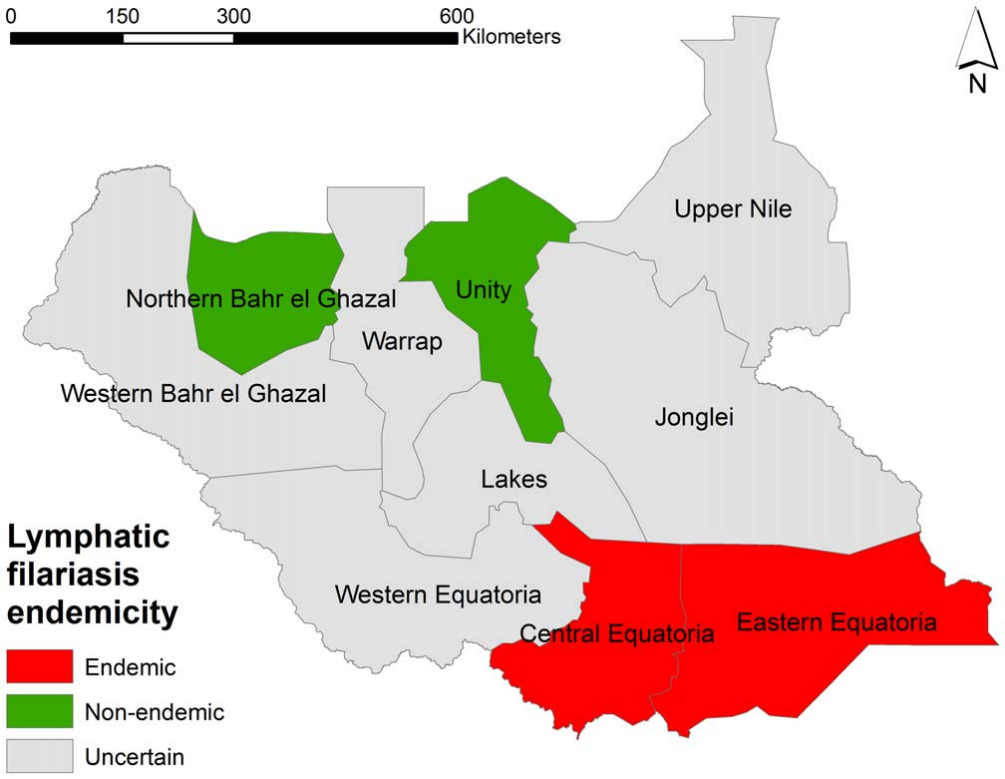
Map showing LF endemic, non-endemic and unmapped areas of South Sudan.

In Central- and Eastern Equatoria states, the proportion of ICT positive individuals ranged from 0.5% to 8.2% by county. In 11 out of the 14 counties in the two states the proportion of ICT positives was above 1%. Using the MDA threshold of ≥1% LF infection prevalence recommended by WHO [2], 11 counties were identified as eligible for treatment. Within the eligible counties an estimated 1.33 million individuals will need to be treated with ivermectin once a year (Table 5 and Figure 3).

**Table 5:** Counties eligible for MDA of PCT to eliminate LF.

State	Infection prevalence ≥1%		
	Counties	Population at risk	Tx's IVM to cover target population^a^
Unity	0	0	0
Central Equatoria	4	859,593	716,475
Eastern Equatoria	7	799,965	639,972
Totals	11	1,659,558	1,327,645

a Tx's = Treatments; IVM  =  Ivermectin; the target population is approximately 80% of population at risk; tablets per treatment of ivermectin are calculated by height, e.g. 1 tablet given to a child between 90 and 119 cm, four tablets for an adult >159 cm etc. with an average dose of three tablets per treatment across the target population.

Twenty of the individuals tested for LF also responded with ‘Yes’ when asked about having experienced or noticed *L. loa* worms moving along the white part of their eyes. However, only two of these individuals reported that this experience did not exceed seven days, and were therefore considered as having a history of eye worm. Both of these were resident in Unity State.

## Discussion

The present study is the largest NTD survey conducted in South Sudan to date and identified large areas of the country as endemic for one or more of the surveyed NTDs. Nearly the entirety of Unity State was identified as endemic for schistosomiasis, while both Central- and Eastern Equatoria states were found largely endemic for all three NTDs surveyed. The predominant STH species was hookworm, which exhibited a distinct geographical distribution, with prevalence greatest in the southern states and lowest in Unity State where thermal exclusion is assumed to limit transmission. There was no evidence of significant *L. loa* transmission, which is consistent with historical reports [13], [14] and preliminary findings of recent RAPLOA surveys conducted by APOC, both indicating that loiasis is by and large confined to Western Equatoria state [4].

Given that South Sudan is a vast and ecologically diverse region, the observed variation in NTD prevalence between and within states was not surprising and is consistent with anecdotal evidence and data from smaller studies [1], [4]. The inherently focal nature of schistosomiasis, compared with STH and LF, has been well documented in other settings [9], [15], [16] and was confirmed again here, particularly in Eastern Equatoria. The purpose of the present mapping was to detect this suspected variation in endemicity for all of the diseases surveyed and classify administrative areas for MDA of PCT intervention. It should be noted, however, that prevalence values presented for schistosomiasis infection (i.e. infected with either or both S. haematobium or S. mansoni) should be viewed as conservative estimates, as not all individuals returned both stool and urine samples and thus not all enrolled individuals could be tested for both species.

The present study had a number of limitations, most of which were identified during our previous survey in Northern Bahr-el-Ghazal [3]. These included the potential biases of purposive sampling, which was adopted to maximise detection of ongoing transmission, and the effect of recent population migration. Accurate boundaries and population figures were not available for many payams, which created difficulties in the selection of survey sites, attributing survey data to a specific administrative area, and prevented us from developing detailed maps indicating schistosomiasis and STH treatment needs at payam level. In addition, the present study was more affected by sporadic insecurity and seasonal inaccessibility, preventing survey teams from assessing one county, Panyijar in Unity State, and several payams, and thus not allowing classification as to whether they are eligible for MDA. Lastly, recent political changes in South Sudan have led to a reorganisation in the number and shape of administrative areas and resulted in inconsistencies in payams between official census information and local government authorities.

## Conclusion

The present survey provided further evidence that rapid mapping to target PCT delivery is an important public health endeavour due to the marked spatial variation of NTD endemicity and the resulting need for evidence-based targeting of treatments. Across the three surveyed states, the distribution and prevalence of major NTDs, in particular schistosomiasis, varied considerably. By sampling accessible payams for STH and schistosomiasis, and counties for LF, we were able to identify areas inhabited by approximately 1.2 and 1.4 million individuals that are eligible for regular MDA with PCT to treat STH and schistosomiasis, respectively, while counties inhabited by a total of about 1.3 million individuals in Central- and Eastern Equatoria states were identified as requiring annual PCT to eliminate LF. The challenge now remains to complete NTD mapping in the remaining states in the country, and to regularly provide treatment to eligible populations.
